# Near-Infrared Dyes: Towards Broad-Spectrum Antivirals

**DOI:** 10.3390/ijms24010188

**Published:** 2022-12-22

**Authors:** Kseniya A. Mariewskaya, Maxim S. Krasilnikov, Vladimir A. Korshun, Alexey V. Ustinov, Vera A. Alferova

**Affiliations:** 1 Shemyakin-Ovchinnikov Institute of Bioorganic Chemistry, Miklukho-Maklaya 16/10, 117997 Moscow, Russia; 2 Department of Chemistry, Lomonosov Moscow State University, Leninskie Gory 1-3, 119991 Moscow, Russia

**Keywords:** NIR dyes, ^1^O_2_ generators, antiviral activity, broad-spectrum antivirals, photosensitizers

## Abstract

Broad antiviral activity in vitro is known for many organic photosensitizers generating reactive oxygen species under irradiation with visible light. Low tissue penetration of visible light prevents further development of antiviral therapeutics based on these compounds. One possible solution to this problem is the development of photosensitizers with near-infrared absorption (NIR dyes). These compounds found diverse applications in the photodynamic therapy of tumors and bacterial infections, but they are scarcely mentioned as antivirals. In this account, we aimed to evaluate the therapeutic prospects of various NIR-absorbing and singlet oxygen-generating chromophores for the development of broad-spectrum photosensitizing antivirals.

## 1. Introduction

Among pathogens causing dangerous viral diseases are many enveloped viruses, such as airborne viruses (e.g., influenza and coronaviruses) and bloodborne viruses (e.g., HCV and HIV). Their characteristic feature is the presence of an outer lipid envelope decorated with membrane proteins. Organic dye-photosensitizers capable of the photogeneration of singlet oxygen (^1^O_2_) and other reactive oxygen species (ROS) often show activity against enveloped viruses [1,2,3,4,5,6,7,8,9,10,11,12,13,14,15,16].

**Figure 1:**
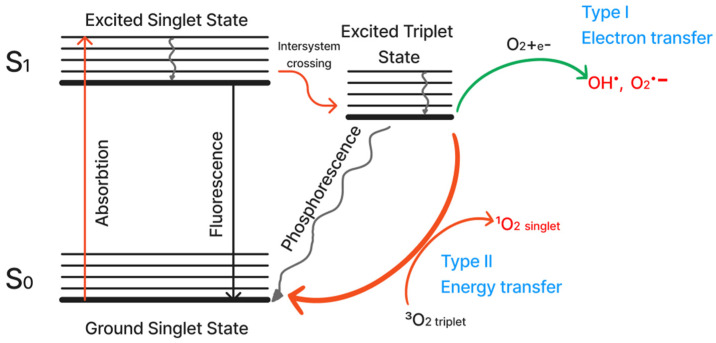
Singlet oxygen generation mechanism (Jablonski diagram) [9,11,16].

The commonly accepted dye-mediated mechanism of ^1^O_2_ and ROS photogeneration is shown in Figure 1. When a molecule is irradiated by a quantum of light, electrons from the ground level transition to the excited S_1_ level without changing spin. In addition to a radiative transition back to the unexcited state, called fluorescence, photosensitizers are able to transition to the more stable excited triplet state, which has about three orders of magnitude longer lifetime than the excited singlet state, since direct relaxation (called phosphorescence) is prohibited. The lifetime of the excited triplet state is sufficient for a dye molecule to collide with molecular oxygen (whose ground state is triplet) and, due to the reorientation of spin states, lead to the formation of two molecules already in singlet states, one of which is singlet oxygen. This transition is called a type II photochemical process, but direct transfer of an excited electron from the triplet level is also possible, resulting in the formation of active oxygen forms, which can also destroy various biomolecules [17,18]. This way of ROS formation is called a type I process.

The wide range of activity of such photosensitizers originates from a target common to all enveloped viruses, their outer lipid membrane. The dye binds to the lipid bilayer due to its special structure; the non-polar core of the molecule intercalates directly into the viral membrane, while the polar parts attach to charged phosphates on the surface. The mechanism of action of these compounds is related to the photogeneration of ^1^O_2_ that oxidizes unsaturated lipids in the viral envelope [19]. Virions with a damaged envelope are unable to fuse with cells [20], so photosensitizing antivirals act as fusion inhibitors. Another advantage of the photodynamic inactivation of virions is that it does not cause viral resistance [21] because the lipid envelope originates from host cells and is not encoded in any way in the viral genome [22].

**Figure 2:**
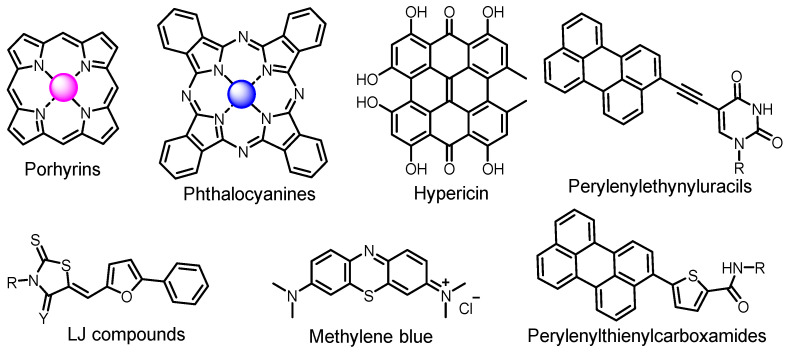
Main scaffolds of antiviral photosensitizers.

The main types of chromophores of such compounds are shown in Figure 2; these are porphyrins and phthalocyanines (usually as metal complexes) [10,23], hypericin [7], perylene compounds [6,7], compound LJ001 and congeners [20], and methylene blue [24]. They are lipophilic aromatic compounds, capable of penetrating into the lipid bilayer. Obvious prerequisites for a pronounced antiviral effect are (1) localization of the chromophore in close proximity to the double bonds of unsaturated fatty acids in the lipid membrane; (2) the presence of oxygen; and (3) light exposure in the area of chromophore absorption. The latter condition is easily met in the case of viral skin infection and can be implemented for the upper respiratory tract [25], which makes many classes of photosensitizers potentially applicable to the therapy of such infections. However, in the case of the internal localization of viral replication foci, light delivery is difficult.

**Figure 3:**
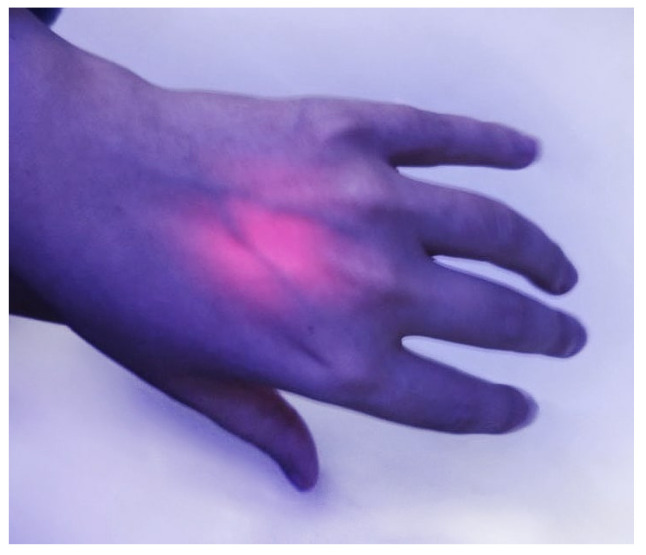
Penetration of near-IR light through tissues; illumination with a 650 nm LED light source.

To solve this problem, several options were proposed, ranging from placing a light source inside the body using various medical devices to introducing, together with a photosensitizer, an auxiliary molecule that emits electromagnetic radiation of the desired wavelength through various chemiluminescent processes. Moreover, the damaging effect of electromagnetic radiation on tissues unrelated to the photosensitizer should be taken into account. Each wavelength range has its own targets in viruses and cells [26], but, in general, it can be noted that, due to lower quantum energy and lower absorption by biomolecules, NIR light itself has a minimal damaging effect compared to the visible and UV ranges. Thus, the simplest and most elegant approach seems to be the use of photosensitizers capable of generating reactive oxygen species when irradiated with electromagnetic radiation in the so-called “therapeutic windows” of 650–900 and 1000–1350 nm [27,28,29], in which tissue transparency is substantially higher (Figure 3) than in the visible range [30,31,32,33].

**Figure 4:**
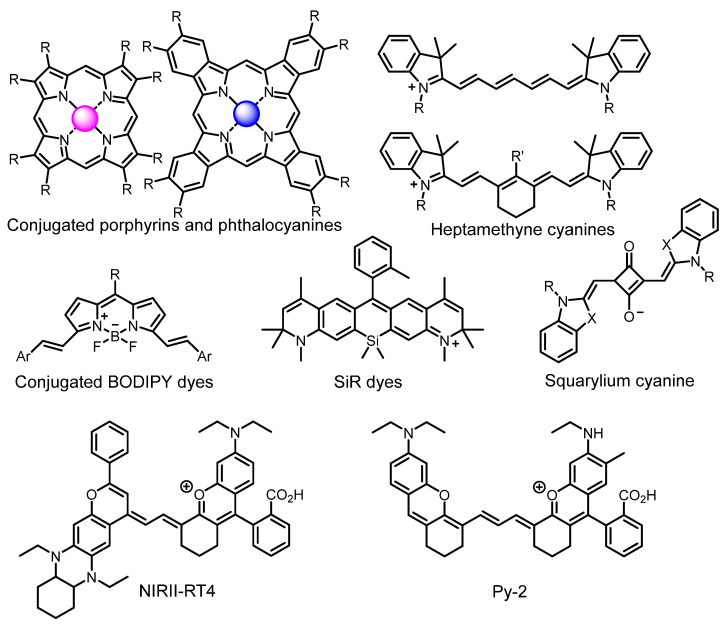
The main types of NIR dyes.

Therefore, such compounds called near-infrared dyes (NIR dyes) are widely used in various fields for imaging/therapeutics/PDT of tumors [34,35,36,37,38,39,40,41,42,43] and bacterial infections [44,45,46] (Figure 4). The development of new NIR dyes is a hot topic [47,48,49,50,51,52,53] that is extensively reviewed [54,55,56]. From the structural point of view, NIR photosensitizers should, on the one hand, have an extended conjugated system reducing the energy difference between LUMO (lowest unoccupied molecular orbital) and HOMO (highest occupied molecular orbital) (corresponding to the difference between levels S_0_ and S_1_ on the Jablonski diagram), thus providing long-wave absorption [57], and, on the other hand, contain a heavy atom generating singlet oxygen [58]. The transition energy from triplet to singlet state for oxygen corresponds to the 1270 nm wavelength [59,60,61], thus, NIR dyes are capable of generating singlet oxygen [62] from a single-photon absorption process at wavelengths of up to 1050 nm [63]. The main classes of ROS-generating NIR dyes are porphyrins and porphyrinoids, phthalocyanines, cyanines, and BODIPYs with an extended π-system [64,65].

Despite the considerable attention that NIR dyes have attracted as agents for PDT, their use for virus inactivation is far less common. Nevertheless, examples of antiviral activity of the NIR dyes summarized in this review show promise for their application to PDT. The aim of this review was to identify structural types of NIR dyes with potential for use in photodynamic virus inactivation. In this work, we limited ourselves to low-molecular-weight organic compounds without considering biopolymers, polymers [66,67,68], nanoparticles [69,70,71], and other NIR-absorbing and singlet oxygen-generating compounds and conjugates [16,72,73,74] that have been proposed for PDT, including viral infections [75], in recent years.

## 2. Antiviral NIR-Photosensitizers

Our first aim was to summarize the infrequent references to the use of NIR dyes as antiviral agents. At present, the most investigated and widely used antiviral photosensitizer is the methylene blue dye. This dye is used as the active ingredient in the THERAFLEX-MB plasma system [76], effectively inactivating the pathogens in blood products [77,78,79]. Its efficacy against SARS-CoV-2 has also been reported [12].

Methylene blue has been proven safe for humans after long-term use in the treatment of methemoglobinemia [80]. It is known that methylene blue binds to DNA; as well as that it can enter both types I and II photochemical processes. Direct electron transfer and the resulting reactive oxygen species (a type I process) likely lead to DNA strand breaks in the absence of oxygen or at low oxygen concentrations. In the presence of oxygen, photo-oxidation occurs according to the type II mechanism; this was proved by the formation of 8-hydroxyguanine in nucleic acids during photo-treatment with methylene blue [80,81]. In addition, methylene blue showed sufficiently high activity against enveloped viruses: SINV, HCV, BVDV, and SARS-CoV-2 (Table 1).

Porphyrins and their analogs are attractive scaffolds for virus inactivation [10]. Chlorin E6 [82], a porphyrin-based dye with the commercial name Talaporfin, previously approved as a drug for the treatment of lung and esophageal cancer by photodynamic therapy, also showed antiviral activity against SARS-CoV-2 [83].

The next class of IR-photosensitizers with antiviral activity are zinc phthalocyanine complexes. It has been shown previously that phthalocyanines containing a zinc atom have the highest antiviral activity among similar complexes with magnesium, transition metals, and metal-free phthalocyanine [9]. All phthalocyanines presented below have IC_50_ in the submicromolar range. Additionally, commercially available IRDye700DX was found to be effective for HIV-1 inactivation in the form of conjugates with an anti-HIV antibody [84,85].

The table below shows substances with maximum absorption within the “therapeutic window” possessing inhibitory activity against one or more viruses.

**Table 1:** Antiviral NIR photosensitizers.

**#**	Scaffold	Compound	Antiviral Activity	λ_abs_ (nm)	References
**1**	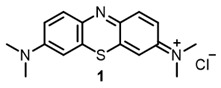		EC_50_: 0.22 ± 0.07, 0.30 ± 0.03 (SARS-CoV-2) μM; TCID_50_: 3.15 (HCV), 4.50 ± 0.66 (BVDV), 5.67 (SINV)	668	[86] (SINV), [87] (HCV, BVDV), [88,89] (SARS-CoV-2)
**2, 3**	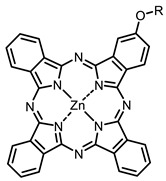	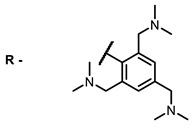	IC_50_: 0.001 nM (H1N1), 0.53 nM (HSV1)	673	[90]
		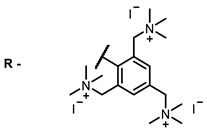	IC_50_: 0.087 nM (H1N1), 0.97 nM (HSV1)	673	
**4, 5**	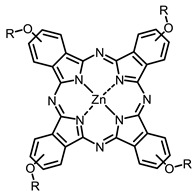	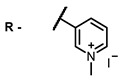	Δlog (gap virus titer and v + PS, 0.58 μM): 4 (HSV-1), 2.4 (VV), 1.8 (BVDV), 0 (NDV), 0.33 (CoxB1), 0.91 (HAdV5)	674	[91,92]
		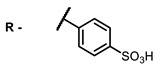	Δlog (gap virus titer and v + PS, 0.64 μM): 4 (HSV-1), 2.2 (VV), 5.3 (BVDV), 1.25 (NDV)	680	
**6, 7, 8**	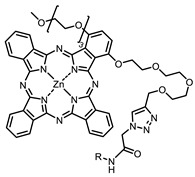	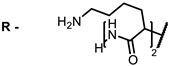	IC_50_: 0.17 nM (H1N1), 0.46 nM (HSV1)	690	[93]
		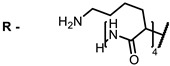	IC_50_: 0.11 nM (H1N1), 0.79 nM (HSV1)	691	
		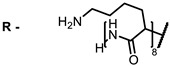	IC_50_: 0.05 nM (H1N1), 0.05 nM (HSV1)	690	
**9**	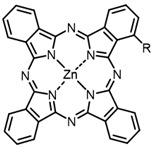	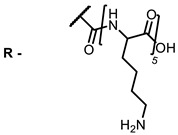	EC_50_: 60.2 nM (SARS-CoV-2)	678	[82,94]
**10**	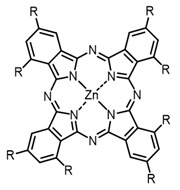	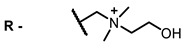	IC_50_: 0.087 nM (H1N1)	675	[95,96]
**11**	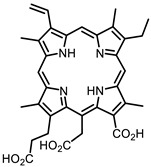		EC_50_: 141 nM (SARS-CoV-2)	654	[82,97]
**12**	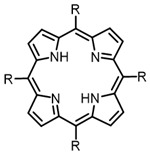	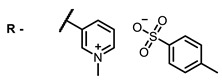		760	[98]

Table 1 shows that reported cases of antiviral activity for NIR dyes are quite rare. At the same time, there are no examples of antiviral dyes with absorbance in the >700 nm region. Nevertheless, rather high values of antiviral activity (in the subnanomolar range) were observed for many of the compounds studied. Similarly to photosensitizers with absorption in the visible range [6], NIR-dyes exhibit broad-spectrum antiviral activity. The affected virus types include +ssRNA (*Flaviviridae*, *Togaviridae*, *Coronaviridae*, *Pricornaviridae*), -ssRNA (*Orthomyxoviridae*, *Paramyxoviridae*), and dsDNA (*Herpesviridae*, *Poxviridae*, *Adenoviridae*) viruses. The vast majority of the susceptible viruses are enveloped (with the exception of coxsackievirus and adenovirus [91,92]).

While studies on the antiviral activity of NIR-absorbing dyes can be called scarce, data on their specific mode of antiviral action and molecular targets is almost non-existent. NIR dyes are thought to act by the same mechanism as other antiviral photosensitizers [3]. The mechanism of inactivation by NIR dyes is generally believed, without further experimental confirmation, to consist of damage to the viral envelope by ROS generation (mainly ^1^O_2_). Nonetheless, a detailed study of the molecular mode of action of these compounds can reveal valuable insights for further drug development. For example, structural TEM study of avian influenza virus H5N8 inactivated by a photosensitizer demonstrated loss of surface glycoproteins under treatment with a low concentration of the compound [95]. The “bald” viral particles retained structural integrity but were inactivated. Therefore, envelope proteins can be effectively targeted by photosensitizers, in addition to unsaturated lipids. Singlet oxygen can damage any biomolecules; for example, it mediated damage to nucleic acids by methylene blue [80]. Enveloped viruses are generally significantly more susceptible to ROS damage. Although all viral components can be a potential molecular target for ROS, proteins and unsaturated lipids of the viral envelope are the most readily available ones [95].

One of the main problems of using NIR dyes as antiviral drugs is their solubility. The dye must contain both a conjugated nonpolar fragment for near-infrared absorption and intercalation into a nonpolar lipid bilayer and polar fragments for more stable fixation in the membrane and increased solubility in water. Unfortunately, at present, the solubility of antiviral photosensitizers in water is low and does not increase upon extension of the non-polar π-system in an attempt to create longer wavelength dyes. The introduction of a constant charge into the molecule can help overcome this problem. There are numerous examples of charged photosensitizers with water solubility suitable for therapeutic applications tested for photodynamic therapy, including NIR dyes [99]. Cationic photosensitizers are believed to be more efficient for antimicrobial PDT; the positive charge allows them to bind to the negatively charged bacterial membranes [90,91]. Data on antiviral activity of charged photosensitizers is rather scarce; there are no clear trends in the structure−antiviral activity relationship. Nonetheless, works in PDT of cancer show that charge variation affects solubility, bioavailability, cellular uptake, intracellular localization, penetration, and excretion rates [100]. Further development of antiviral photosensitizers can be based on data on the cytotoxic properties of the dyes and approaches to their tuning by structural variation.

When discussing biological activity, it is important to note the cytotoxicity of various dyes. Most often, this is not a problem, since the antiviral activity of the dyes is so high that it exceeds the toxicity of the molecule by order of magnitude. A good example is cyanine dyes [101]. One plausible explanation for this tendency is the extracellular mode of antiviral action for NIR-dyes, combined with a generally high molecular weight. The expanded π-system required for long-wavelength absorption leads to a significant increase in molecular weight. Bulky hydrophobic dyes tend to have low cellular uptake, leading to low dark cytotoxicity, whereas virus inactivation does not require membrane penetration and takes place extracellularly.

The potential cytotoxicity of metal complexes not only as photosensitizers but also as heavy metal ion sources, should be always taken into account. Fortunately, metal complexes are currently massively studied as potential antibiotics [102,103,104,105], thus giving large datasets on their cytotoxicity.

It is also worth noting that, for photosensitizers, a correct assessment of both activity and cytotoxicity is a methodologically difficult task. The observed biological effect is influenced by many parameters that are not controlled by standard methods. For example, these parameters include the duration and intensity of irradiation, the match between the irradiation wavelength and the dye absorption bands, oxygen concentration in the medium, and oxygen access under different incubation conditions. Under such conditions, there can be significant distortions in the results and low reproducibility. The biological effect of dyes with absorption maxima far from the visible region can be markedly underestimated due to less intense irradiation. Effective investigation of photosensitizer-based drugs requires developing activity verification protocols that take into account the peculiarities of this class of antivirals. Classical approaches for transitioning from in vitro testing to testing on in vivo models also need significant adjustments.

## 3. ^1^O_2_ Generators

As mentioned earlier, the main requirements for a molecule to be a potential effective broad-spectrum NIR antiviral drug are direct absorption in the near-infrared region and an acceptable quantum yield of singlet oxygen generation. Recently, the high interest in NIR dyes for PDT has led to a large amount of data on the photophysical and photochemical properties and ROS generation ability for a wide range of structural types of dyes. A dye molecule in the excited triplet state can interact with oxygen from the air to form singlet oxygen. To identify the most promising structures in PDT for viruses, we summarized photosensitizers possessing an absorption maximum at >630 nm and high quantum yield of singlet oxygen (Φ**_Δ_** > 0.1), which plays a key role in the antiviral activity of photosensitizers (Table 2).

To detect singlet oxygen and estimate photosensitizer parameters, the quantum yield of singlet oxygen is measured by its own weak phosphorescence [106], by EPR spectroscopy in the course of oxidation of secondary amines to stable radicals [107], and using various chemiluminescent, chromogenic, and fluorogenic probes [108,109,110]. Oxygen generation of all the structures we have considered is evaluated with a special indicator, the most common of which is 1,3-diphenylisobenzofuran (DPBF) [111]. In the presence of singlet oxygen, DPBF is rapidly oxidized, and accordingly, the intensity of its absorption decreases.

**Table 2:** Singlet oxygen generators.

**#**	Scaffold	Compound	λ_abs_ (nm)	Φ_Δ_ *	References
**1**	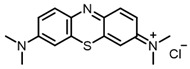		668	0.52	[112]
**2**	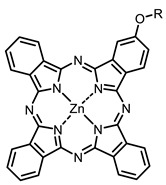	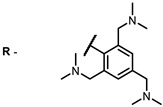	673	0.54	[90]
**3**		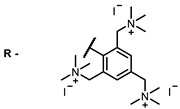	673	0.63	
**4**	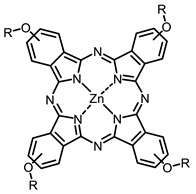	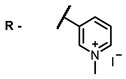	674	0.41	[91]
**5**		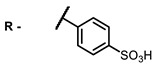	680	0.55	
**6**	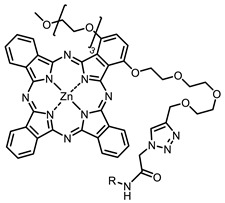	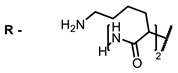	690	0.86	[93]
**7**		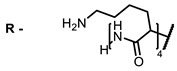	691	0.89	
**8**		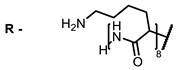	690	0.86	
**9**	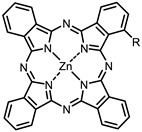	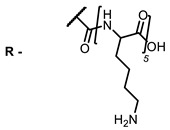	678	0.63	[113]
**10**	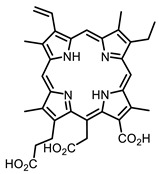		654	0.75	[97]
**11**	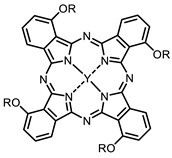	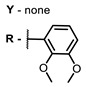	694, 722	0.18	[114]
**12**		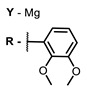	694	0.34	
**13**		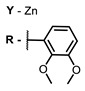	698	0.57	
**14**		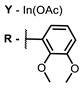	705	0.66	
**15**	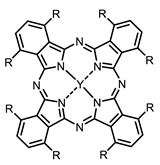	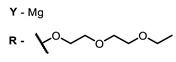	739	0.30	[115]
**16**		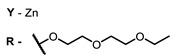	740	0.47	
**17**		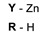	672	0.67	
**18**	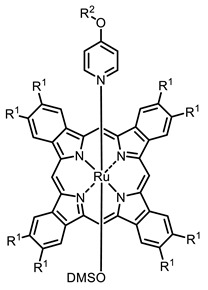	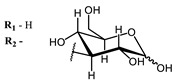	637	0.99	[116]
**19**		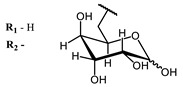	638	0.95	
**20**		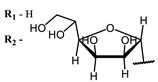	633	0.8	
**21**		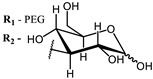	643	0.74	
**22**		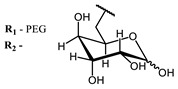	643	0.74	
**23**		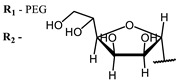	630	0.52	
**24**	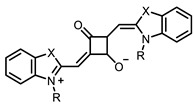	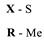	647	0.17	[117]
**25**		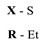	650	0.26	
**26**		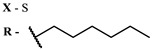	650	0.26	
**27**		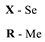	662	0.31	
**28**		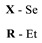	665	0.31	
**29**		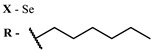	668	0.31	
**30**		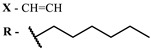	710	0.13	
**31**	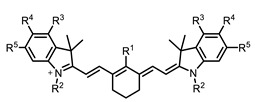	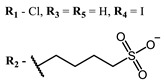	790	0.66	[102,103,107,109,110,114,118]
**32**		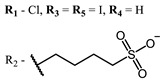	687	0.44	
**33**		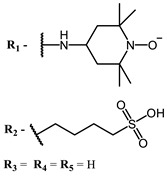	692	0.17	
**34**		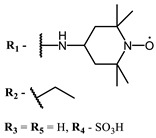	660, 790	0.2	
**35**		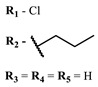	785	0.13	
**36**		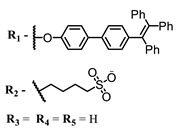	781	+	
**37**		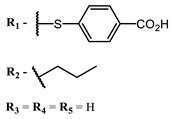	806	+	
**38**		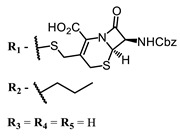	810	+	
**39**		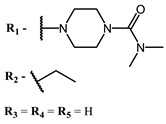	686	0.11	
**40**		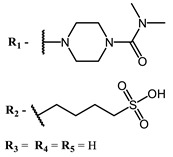	687	0.07	
**41**	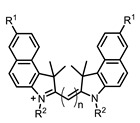	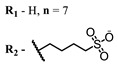	780	0.08	
**42**		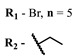	685	+	[119]
**43**		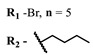	688	+	
**44**	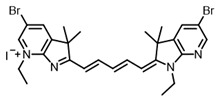		736	0.03	[120]
**45**	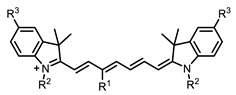	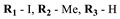	773	0.2	[121]
**46**		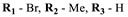	736	0.04	
**47**		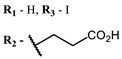	780	0.75	[122]
**48**	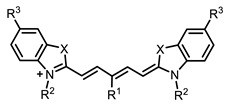	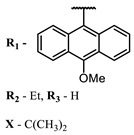	650	0.11	[123]
**49**		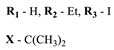	668	0.17	
**50**		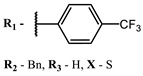	666	0.17	[124]
**51**		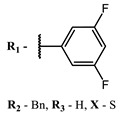	663	0.2	
**52**		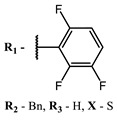	655	0.39	
**53**	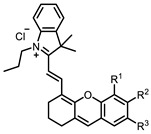	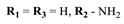	700	0.12	[125]
**54**		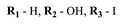	715	0.22	
**55**		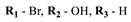	715	0.21	
**56**		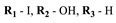	720	0.8	
**57**	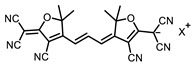	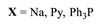	647	+	[126]
**58**	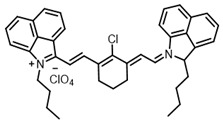		1040	+	[63]
**59**	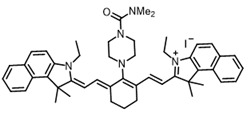		693	0.12	[114]
**60**	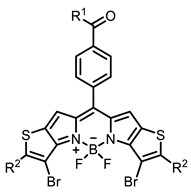	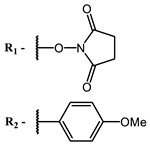	665	0.76	[127]
**61**		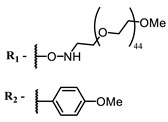	665	0.59	
**62**	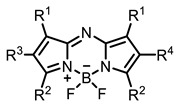	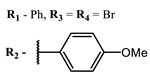	679	0.74	[128]
**63**		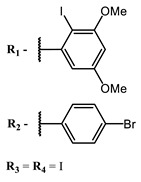	666	0.70	[129]
**64**		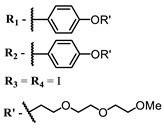	670	0.88	[130]
**65**		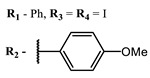	679	0.24	[120]
**66**		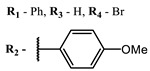	679	0.1	[120]
**67**		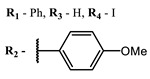	678	0.52	[120]
**68**		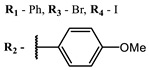	679	0.29	[120]
**69**		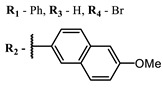	730	+	[128]
**70**		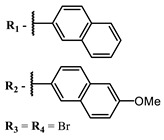	698	+	[129]
**71**		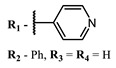	667	0.62	[131]
**72**		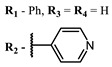	638	0.89	[131]
**73**	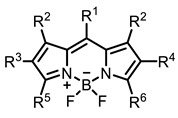	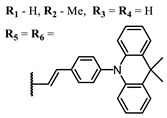	643	0.29	[132]
**74**		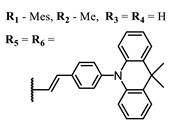	633	0.23	
**75**		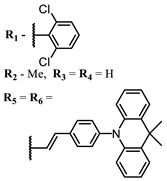	648	0.31	
**76**		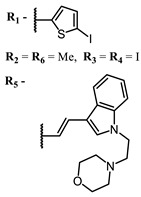	660	0.44	[133]
**77**		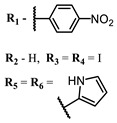	701	0.63	[123]
**78**		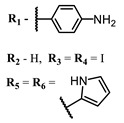	668	0.69	[123]
**79**		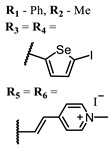	671	0.32	[131,134]
**80**		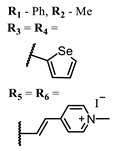	663	0.17	[131,134]
**81**		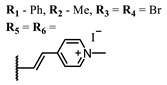	658	0.1	[131,134]
**82**		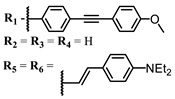	711	0.15	[127]
**83**		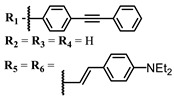	713	0.13	[127]
**84**		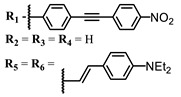	716	0.05	[127]
**85**		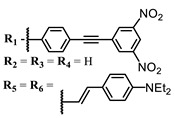	718	0.04	[127]
**86**		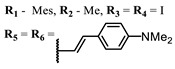	747	0.73	[130]
**87**		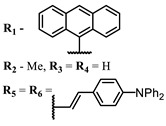	708	0.60	[132]
**88**		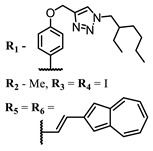	778	0.11	[133]
**89**	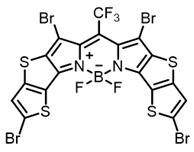		750	0.41	[135]
**90**	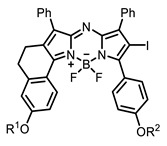	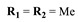	677	0.51	[136]
**91**		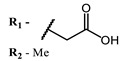	677	0.25	
**92**		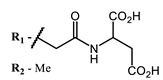	679	0.61	
**93**		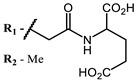	678	0.63	
**94**		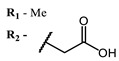	678	0.71	
**95**		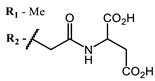	675	0.66	
**96**		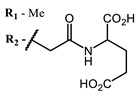	676	0.69	
**97**	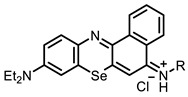	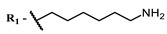	670	0.68	[137]
**98**		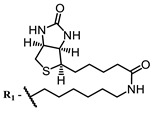	670	0.69	
**99**	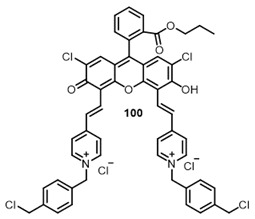		640	0.5	[138]
**100**	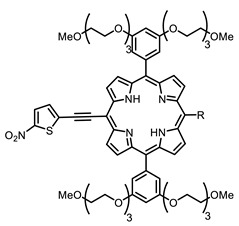	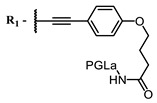	708, 782	0.35	[139]
**101**		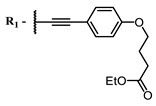	712, 786	0.18	
**102**	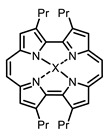	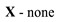	653	0.18	[140]
**103**		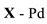	~685	0.51	
**104**	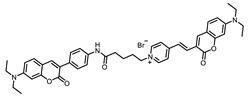		840	0.85	[141]
**105**	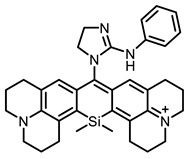		710	+	[142]

* “+” corresponds to qualitative singlet oxygen generation results.

Based on the data in the table, the following conclusions can be made. Porphyrins, phthalocyanines, cyanines, and BODIPY are the most studied classes of IR dyes in terms of ROS generation.

On average, phthalocyanines exhibit rather high quantum yields of singlet oxygen (0.4–0.9). Quantum yield is significantly affected both by the presence of metal in the complex and the introduction of substituents into the phthalocyanine core. The highest quantum yields of singlet oxygen with significant quenching of fluorescence were observed for compounds **6**–**8** (Φ_Δ_ 0.86–0.89) as a result of their di-α-substitution [93]. The introduction of substituents into phthalocyanine molecules, in addition to optimizing their photophysical properties, can serve to improve their solubility, which is very important for both in vitro and in vivo applications. For example, the introduction of (Lys)_5_ (oligolysin) residues improved the water solubility of the ZnPc conjugate [113]. For some of the phthalocyanines, an association between antimicrobial properties and ROS generation under red light irradiation has been shown [90].

Squarylium cyanines with a heavy atom of selenium in the “indolenine” parts **27**–**29** show higher values of ^1^O_2_ quantum yield than their analogs with sulfur [117].

Cyanines with a heavy atom in the “core” have significantly lower values of ^1^O_2_ quantum yield than those with iodine or bromine in the indole and/or indolenine part. An increase in the number of heavy atoms (more than two) in a cyanine molecule leads to a decrease in quantum yield of singlet oxygen [118]. Also, cyanines **33**–**34** with TEMPO in a central fragment of their structure have good enough values of ^1^O_2_ quantum yield, higher than close compounds **39**, **40,** and **59** with piperazine [143,144,145]. The insertion of a heavy atom into the cyanine nucleus is less effective for increasing ROS generation than insertion into the indolenine part [121]. Interesting experimental results on the influence of the nature of the counterion were obtained for cyanine derivative **55**: only C3T-Pc with a bulky phosphonium counterion can form supramolecular *J*-aggregates in aqueous solutions, leading to significantly red-shifted emission and enhanced Φ_Δ_ [126].

The introduction of heavy atoms or reactive groups into BODIPY significantly increases singlet oxygen generation, but the introduction of more than two heavy atoms into the molecule negatively affects this value. BODIPY **61**, with two atoms of bromine, an extended π-system, and a very long hydrophilic PEG-group, is a very interesting compound: it is a good singlet oxygen generator and, due to its structure, may be safer for humans than other compounds of this class [127]. Compound **64** has ultrahigh quantum yield of singlet oxygen (Φ_Δ_ 88%), thus enabling a proof-of-concept application of highly-efficient PDT in vivo under ultralow near-infrared light power density [130]. A very interesting article is devoted to the study of the influence of various heavy atoms and their number in a molecule on quantum yield singlet oxygen [146]. Compound **67** has only one atom of iodine and higher Φ_Δ_ than similar compounds with two atoms of iodine, and one and two atoms of bromine. However, compounds **62**–**63** and **76**–**78**, **89** have two, three, and four heavy atoms, respectively, in their structures and high quantum yields [128,129,133,135,147]. Thus, we cannot make an unambiguous assessment of what number of heavy atoms in the structure of a BODIPY provides maximum singlet oxygen generation. The presence of dimethylacridine fragments in the structure of the compound leads to an increase in the singlet oxygen generation, but not as large as heavy atoms, such as iodine [105]. High quantum yields of singlet oxygen were also achieved for heavy-atom-free BODIPY dyes, e.g., **71** and **72** demonstrate high singlet oxygen (^1^O_2_) generation efficiency (up to 0.85–0.89) [148]. The presence of electron donor groups conjugated with the π-system in the molecule was found to increase Φ_Δ_ [149]. Expansion of the π-system from phenyl to polyaromatic substituents does not result in either a shift to the IR region or an increase in ROS generation, but presumably increases toxicity in the dark [150,151,152,153,154]. Glytamic acid-derived aza-BODIPY **96** has good water solubility and high ROS generation. The presence of an amide group in the ring located close to the iodine atom contributes to this effect [136]. An association of activity with ROS generation was shown for the antibacterial photosensitizer **79**: the inhibitory effect of this BODIPY on *S. aureus* was not observed when ROS species were scavenged by KI or NaN_3_ [131,134].

Selenium-containing compounds **97** and **98** are promising PSs with their high photostability and ^1^O_2_ quantum yield values, as well as their similarity to methylene blue, which is safe for humans [80,137].

Porphyrins generally exhibit rather low quantum yields of ROS; however, it should be noted that a design of an extended π-conjugated photosensitizer linked to an antimicrobial peptide enabled its excitation in the near IR to perform PDT in the optical therapeutic window. The conjugate has shown good photostability and capacity to generate singlet oxygen [139].

Thus, the most effective way to provide a bathochromic shift is either the introduction of various heterocycles as substituents or the expansion of the π-system of the dye core itself by adding additional aromatic rings. For example, cyanine **58**, BODIPY **60**–**61**, **77**, **78**, and **89**. It should be noted that the absorption maximum for aza-BODIPY is shifted by ~70–80 nm to a redder region than for analogous BODIPYs. Among the various substituents that increase ROS generation, the most effective are iodine atoms. The optimal amount differs for different classes of compounds: while introducing more than two atoms is undesirable for cyanines; in the case of BODIPY, this amount depends on the structural features of a particular compound. The position of the heavy atom in the molecule is also important: in BODIPY these are positions 2 and 6; in cyanines, it is the indolenine ring. The lowest Φ_Δ_ values are detected for the compounds with a heavy halogen atom as the anion. An exception is compound **104** with the bromine anion, which has an extremely high yield of singlet oxygen generation. Phthalocyanines can form complexes with various metal ions, the highest ROS generation is observed in zinc phthalocyanines.

Table 2 shows that the number of NIR dyes capable of generating singlet oxygen, including high yields, is significant. However, NIR dyes are often developed for in vivo imaging and are not studied as ROS generators. Such dyes are an additional source of potential photosensitizers. In addition, for such compounds, the ways to achieve the greatest long-wavelength shift of absorption and fluorescence maxima are well known, so variation of their structures (for example, with the introduction of a heavy atom) is promising for obtaining compounds with optimal properties—long-wavelength absorption and quenched fluorescence.

In addition, it should be noted that a high yield of singlet oxygen generation often leads to low photostability for many of the given compounds due to low oxygen lifetime in the singlet state and its high reactivity, as a result of which the dye itself is oxidized and destroyed by the generated singlet oxygen. In this case, it is worthwhile to additionally measure the photostability of the studied compounds in light and in the dark.

## 4. Conclusions

Despite the fact that the synthesis of dyes with the absorption peak falling within the “therapeutic window” is not new, very few such dyes are currently known and have been studied for the presence or absence of antiviral activity. The compounds considered above are promising for this field of research.

**Figure 5:**
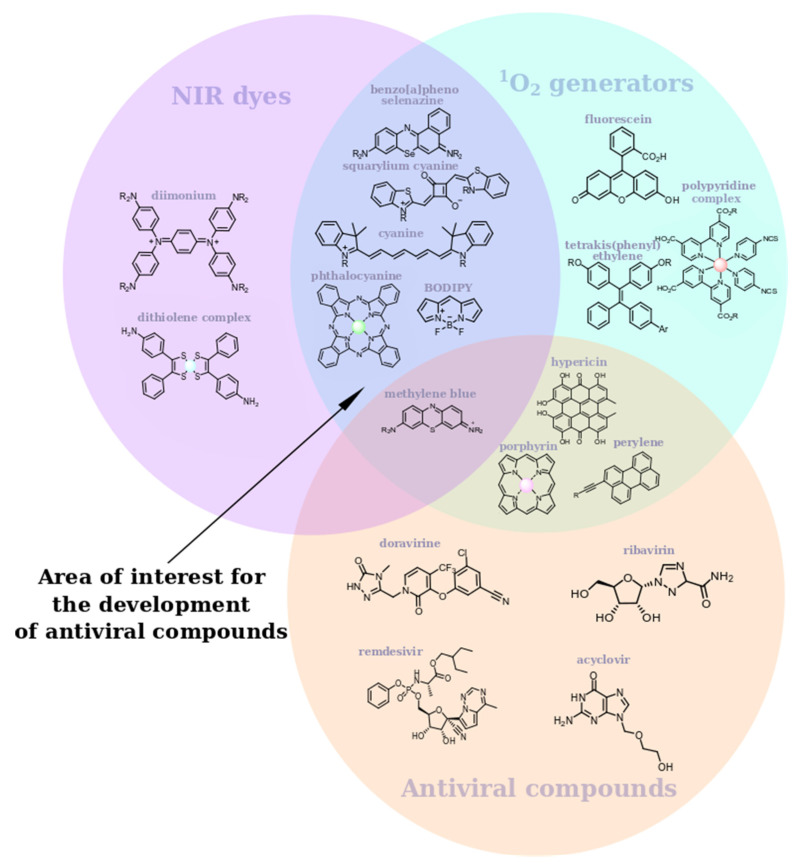
Analysis of prospective antiviral photosensitizing scaffolds.

Currently, NIR dyes are being actively developed as antitumor agents, but, based on the information we analyzed, we can conclude that such structures are very promising for photodynamic inactivation of viruses as well. Thus, we found that all NIR dyes with proven antiviral properties are singlet oxygen generators. At the same time, there are many NIR dyes with well-studied singlet oxygen generation ability which have never been studied as antiviral compounds (Figure 5). The search for new antiviral photosensitizers with absorption in the IR region is the most promising among such scaffolds.

By analyzing the collected photophysical and antiviral properties of NIR dyes, we can identify general patterns in their structural design. The dye molecule must contain an extensive conjugate structure in order to shift absorption into the NIR region and freely intercalate into the viral membrane, and a polar part or polar substituents that increase the water solubility of the molecule and promote a more stable attachment to the lipid membrane of enveloped viruses through interaction with the polar ends. To increase quantum yield of singlet oxygen, one or two heavy atoms should be introduced into the molecule to quench the fluorescence, preferably directly into the dye core, not into the linker. Also, if there is no rigid fixation of the π-system, quantum yield of the fluorescence drops, which often leads to improved singlet oxygen generation [155].

Further development of antiviral compounds based on these scaffolds is attractive for several reasons. First, photosensitizers generally have a wide spectrum of antiviral activity, as demonstrated by photosensitizers based on perylene, hypericin [7], phenothiazine, porphyrin, and phthalocyanine [9]. Secondly, dyes with an absorption maximum falling within the “therapeutic window” require electromagnetic radiation capable of penetrating tissues for their excitation. Third, the high quantum yield of singlet oxygen makes it possible to expect high antiviral activity for such compounds. Nevertheless, there are some notable difficulties in the study of photosensitizers. Correct study of their activity and cytotoxicity requires a modification of standard techniques to control the intensity, wavelength, and dose of irradiation on all stages of research. As for in vivo tests, even in the case of NIR dyes, selection of suitable models and the development of drug forms, administration methods and experiment protocols with irradiation dose control is a challenge. On the other hand, the wide spectrum of activity and ultra-low effective doses of antiviral photosensitizers provide potential for effective drugs. Ultra-low active concentrations of photosensitizers are achieved due to the fact that they are not directly acting damaging agents. A huge amount of oxygen is dissolved in the target environment, and the photosensitizer can convert it to an active singlet form over many cycles (up to several million) of excitation−relaxation within the bounds of its photostability.

Scaffolds of cyanine and BODIPY NIR-dyes are of particular interest. Cyanine dyes and BODIPY dyes have been very well studied, and various methods for the synthesis and modification of their derivatives have been developed. As can be seen from the data presented in Table 2, the quantum yield of singlet oxygen for these compounds is often very high. In addition, low cytotoxicity is observed for the members of these structural families. It is also worth noting that BODIPY dyes and cyanine dyes, currently not yet fully investigated from this point of view, are of increasing interest as a basis for obtaining potentially active antiviral substances with NIR-range absorbance. There are already known cases of antiviral activity for derivatives of substances in these classes with absorption in the visible range. For example, the visible-range absorbing cyanine dye lumin showed antiviral activity [156], and a BODIPY-based dye (λ_max_ (H_2_O) 509 nm) was described as an antiviral [157]. All this suggests that NIR BODIPYs and cyanines have high potential as photosensitizers for the development of broad-spectrum antiviral therapeutics.

Since NIR singlet oxygen generators have an antiviral effect near the target, the unsaturated lipids of the viral membrane, it may be appropriate to target the viral lipid membrane rather than the cell membrane when developing such antiviral drugs. This can be achieved by conjugation with antibodies against various domains of viral membrane proteins, e.g., a spike protein. After specific delivery to the outer viral membrane, the lipophilic dye on a suitable linker will penetrate the lipid bilayer and generate singlet oxygen there. Moreover, with this kind of delivery, singlet oxygen can also have a damaging effect on viral envelope proteins, additionally inactivating the viral particle.

Modern molecular modeling and simulation techniques could be useful for revealing the photosensitizers’ affinity to and interactions with lipid membrane. For example, such studies were performed for the broad-spectrum antiviral and singlet oxygen photogenerator (however, not NIR-dye) hypericin [158]. A recent in silico study of indocyanine green revealed the receptor-binding domain in SARS-CoV-2 could be a potential binding site for cyanine dye [159].

## Data Availability

Not applicable.

## References

[1] Wainwright M. (2003). Local treatment of viral disease using photodynamic therapy. Int. J. Antimicrob. Agents.

[2] Wainwright M. (2004). Photoinactivation of viruses. Photochem. Photobiol. Sci..

[3] Costa L., Faustino M.A.F., Neves M.G.P.M.S., Cunha Â., Almeida A. (2012). Photodynamic inactivation of mammalian viruses and bacteriophages. Viruses.

[4] Kunstek H., Vreken F., Keita A., Hamblin M.R., Dumarçay F., Varbanov M. (2022). Aspects of antiviral strategies based on different phototherapy approaches: Hit by the light. Pharmaceuticals.

[5] Delcanale P., Abbruzzetti S., Viappiani C. (2022). Photodynamic treatment of pathogens. Riv. Nuovo Cimento.

[6] Mariewskaya K.A., Tyurin A.P., Chistov A.A., Korshun V.A., Alferova V.A., Ustinov A.V. (2021). Photosensitizing antivirals. Molecules.

[7] Alferova V.A., Mikhnovets I.E., Chistov A.A., Korshun V.A., Tyurin A.P., Ustinov A.V. (2022). Perylene as a controversial antiviral scaffold. Medicinal Chemistry of Tick-Borne Encephalitis.

[8] Conrado P.C.V., Sakita K.M., Arita G.S., Galinari C.B., Gonçalves R.S., Lopes L.D.G., Lonardoni M.V.C., Teixeira J.J.V., Bonfim-Mendonça P.S., Kioshima E.S. (2021). A systematic review of photodynamic therapy as an antiviral treatment: Ootential guidance for dealing with SARS-CoV-2. Photodiagn. Photodyn. Ther..

[9] Wiehe A., O’Brien J.M., Senge M.O. (2019). Trends and targets in antiviral phototherapy. Photochem. Photobiol. Sci..

[10] Lebedeva N.S., Gubarev Y.A., Koifman M.O., Koifman O.I. (2020). The application of porphyrins and their analogues for inactivation of viruses. Molecules.

[11] Willis J.A., Cheburkanov V., Kassab G., Soares J.M., Blanco K.C., Bagnato V.S., Yakovlev V.V. (2021). Photodynamic viral inactivation: Recent advances and potential applications. Appl. Phys. Rev..

[12] Mahmoudi H. (2021). Photodynamic therapy as a new technology for inactivation of coronavirus disease (COVID-19). Front. Biomed. Technol..

[13] Almeida A., Faustino M.A.F., Neves M.G.P.M.S. (2020). Antimicrobial photodynamic therapy in the control of COVID-19. Antibiotics.

[14] Wang N., Ferhan A.R., Yoon B.K., Jackman J.A., Cho N.-J., Majima T. (2021). Chemical design principles of next-generation antiviral surface coatings. Chem. Soc. Rev..

[15] Sadraeian M., Zhang L., Aavani F., Biazar E., Jin D. (2022). Photodynamic viral inactivation assisted by photosensitizers. Mater. Today Phys..

[16] Pham T.C., Nguyen V.-N., Choi Y., Lee S., Yoon J. (2021). Recent strategies to develop innovative photosensitizers for enhanced photodynamic therapy. Chem. Rev..

[17] Di Mascio P., Martinez G.R., Miyamoto S., Ronsein G.E., Medeiros M.H.G., Cadet J. (2019). Singlet molecular oxygen reactions with nucleic acids, lipids, and proteins. Chem. Rev..

[18] Halliwell B., Gutteridge J.M.C. (2015). Free Radicals in Biology and Medicine.

[19] Bacellar I.O.L., Oliveira M.C., Dantas L.S., Costa E.B., Junqueira H.C., Martins W.K., Durantini A.M., Cosa G., Di Mascio P., Wainwright M. (2018). Photosensitized membrane permeabilization requires contact-dependent reactions between photosensitizer and lipids. J. Am. Chem. Soc..

[20] Hollmann A., Castanho M.A.R.B., Lee B., Santos N.C. (2014). Singlet oxygen effects on lipid membranes: Implications for the mechanism of action of broad-spectrum viral fusion inhibitors. Biochem. J..

[21] Maisch T. (2015). Resistance in antimicrobial photodynamic inactivation of bacteria. Photochem. Photobiol. Sci..

[22] Vigant F., Santos N.C., Lee B. (2015). Broad-spectrum antivirals against viral fusion. Nat. Rev. Microbiol..

[23] Sadraeian M., Junior F.F.P., Miranda M., Galinskas J., Fernandes R.S., da Cruz E.F., Fu L., Zhang L., Diaz R.S., Cabral-Miranda G. (2022). Study of viral photoinactivation by UV-C light and photosensitizer using a pseudotyped model. Pharmaceutics.

[24] Wainwright M. (2000). Methylene blue derivatives—suitable photoantimicrobials for blood product disinfection?. Int. J. Antimicrob. Agents.

[25] Dias L.D., Blanco K.C., Bagnato V.S. (2020). COVID-19: Beyond the virus. The use of photodynamic therapy for the treatment of infections in the respiratory tract. Photodiagn. Photodyn. Ther..

[26] Sadraeian M., Zhang L., Aavani F., Biazar E., Jin D. (2022). Viral inactivation by light. eLight.

[27] Dąbrowski J.M., Pucelik B., Regiel-Futyra A., Brindell M., Mazuryk O., Kyzioł A., Stochel G., Macyk W., Arnaut L.G. (2016). Engineering of relevant photodynamic processes through structural modifications of metallotetrapyrrolic photosensitizers. Coord. Chem. Rev..

[28] Weissleder R. (2001). A clearer vision for *in vivo* imaging. Nat. Biotechnol..

[29] Smith A.M., Mancini M.C., Nie S. (2009). Second window for in vivo imaging. Nat. Nanotechnol..

[30] Golovynskyi S., Golovynska I., Stepanova L.I., Datsenko O.I., Liu L., Qu J., Ohulchanskyy T.Y. (2018). Optical windows for head tissues in near-infrared and short-wave infrared regions: Approaching transcranial light applications. J. Biophotonics.

[31] Zhang H., Salo D., Kim D.M., Komarov S., Tai Y.-C., Berezin M.Y. (2016). Penetration depth of photons in biological tissues from hyperspectral imaging in shortwave infrared in transmission and reflection geometries. J. Biomed. Opt..

[32] Li C., Chen G., Zhang Y., Wu F., Wang Q. (2020). Advanced fluorescence imaging technology in the near-infrared-II window for biomedical applications. J. Am. Chem. Soc..

[33] Feng Z., Tang T., Wu T., Yu X., Zhang Y., Wang M., Zheng J., Ying Y., Chen S., Zhou J. (2021). Perfecting and extending the near-infrared imaging window. Light Sci. Appl..

[34] Lange N., Szlasa W., Saczko J., Chwiłkowska A. (2021). Potential of cyanine derived dyes in photodynamic therapy. Pharmaceutics.

[35] Okubo K., Umezawa M., Soga K. (2021). Near infrared fluorescent nanostructure design for organic/inorganic hybrid system. Biomedicines.

[36] Li H., Kim Y., Jung H., Hyun J.Y., Shin I. (2022). Near-infrared (NIR) fluorescence-emitting small organic molecules for cancer imaging and therapy. Chem. Soc. Rev..

[37] Ilina K., Henary M. (2021). Cyanine dyes containing quinoline moieties: History, synthesis, optical properties, and applications. Chem. Eur. J..

[38] Zhang X., An L., Tian Q., Lin J., Yang S. (2020). Tumor microenvironment-activated NIR-II reagents for tumor imaging and therapy. J. Mater. Chem. B.

[39] Namikawa T., Fujisawa K., Munekage E., Iwabu J., Uemura S., Tsujii S., Maeda H., Kitagawa H., Fukuhara H., Inoue K. (2018). Clinical application of photodynamic medicine technology using light-emitting fluorescence imaging based on a specialized luminous source. Med. Mol. Morphol..

[40] Pucelik B., Sułek A., Dąbrowski J.M. (2020). Bacteriochlorins and their metal complexes as NIR-absorbing photosensitizers: Properties, mechanisms, and applications. Coord. Chem. Rev..

[41] Yan M., He D., Zhang L., Sun P., Sun Y., Qu L., Li Z. (2022). Explorations into the meso-substituted BODIPY-based fluorescent probes for biomedical sensing and imaging. Tr. Anal. Chem..

[42] Karaman O., Alkan G.A., Kizilenis C., Akgul C.C., Gunbas G. (2023). Xanthene dyes for cancer imaging and treatment: A material odyssey. Coord. Chem. Rev..

[43] Mao Z., Kim J.H., Lee J., Xiong H., Zhang F., Kim J.S. (2023). Engineering of BODIPY-based theranostics for cancer therapy. Coord. Chem. Rev..

[44] Agrawal T., Avci P., Gupta G., Rineh A., Lakshmanan S., Batwala V., Tegos G., Hamblin M. (2015). Harnessing the power of light to treat staphylococcal infections focusing on MRSA. Curr. Pharm. Des..

[45] Meerovich G.A., Akhlyustina E.V., Tiganova I.G., Lukyanets E.A., Makarova E.A., Tolordava E.R., Yuzhakova O.A., Romanishkin I.D., Philipova N.I., Zhizhimova Y.S. (2019). Novel polycationic photosensitizers for antibacterial photodynamic therapy. Adv. Exp. Med. Biol..

[46] Nguyen V.-N., Zhao Z., Tang B.Z., Yoon J. (2022). Organic photosensitizers for antimicrobial phototherapy. Chem. Soc. Rev..

[47] Ren T., Wang Z., Xiang Z., Lu P., Lai H., Yuan L., Zhang X., Tan W. (2021). A general strategy for development of activatable NIR-II fluorescent probes for in vivo high-contrast bioimaging. Angew. Chem. Int. Ed..

[48] Zhang X., Chen Y., He H., Wang S., Lei Z., Zhang F. (2021). ROS/RNS and base dual activatable merocyanine-based NIR-II fluorescent molecular probe for in vivo biosensing. Angew. Chem. Int. Ed..

[49] Gardner S.H., Brady C.J., Keeton C., Yadav A.K., Mallojjala S.C., Lucero M.Y., Su S., Yu Z., Hirschi J.S., Mirica L.M. (2021). A general approach to convert hemicyanine dyes into highly optimized photoacoustic scaffolds for analyte sensing. Angew. Chem. Int. Ed..

[50] Li B., Liu H., He Y., Zhao M., Ge C., Younis M.R., Huang P., Chen X., Lin J. (2022). A “self-checking” pH/viscosity-activatable NIR-II molecule for real-time evaluation of photothermal therapy efficacy. Angew. Chem. Int. Ed..

[51] Qin Z., Ren T., Zhou H., Zhang X., He L., Li Z., Zhang X., Yuan L. (2022). NIRII-HDs: A versatile platform for developing activatable NIR-II fluorogenic probes for reliable in vivo analyte sensing. Angew. Chem. Int. Ed..

[52] He L., He L., Xu S., Ren T., Zhang X., Qin Z., Zhang X., Yuan L. (2022). Engineering of reversible NIR-II redox-responsive fluorescent probes for imaging of inflammation in vivo. Angew. Chem. Int. Ed..

[53] Lan Q., Yu P., Yan K., Li X., Zhang F., Lei Z. (2022). Polymethine molecular platform for ratiometric fluorescent probes in the second near-infrared window. J. Am. Chem. Soc..

[54] Exner R.M., Cortezon-Tamarit F., Pascu S.I. (2021). Explorations into the effect of *meso*-substituents in tricarbocyanine dyes: A path to diverse biomolecular probes and materials. Angew. Chem. Int. Ed..

[55] Lei Z., Zhang F. (2021). Molecular engineering of NIR-II fluorophores for improved biomedical detection. Angew. Chem. Int. Ed..

[56] Mu J., Xiao M., Shi Y., Geng X., Li H., Yin Y., Chen X. (2022). The chemistry of organic contrast agents in the NIR-II window. Angew. Chem. Int. Ed..

[57] Fabian J., Nakazumi H., Matsuoka M. (1992). Near-infrared absorbing dyes. Chem. Rev..

[58] Hintze C., Morgen T.O., Drescher M. (2017). Heavy-atom effect on optically excited triplet state kinetics. PLoS ONE.

[59] Krasnovsky A.A. (1979). Photoluminescence of singlet oxygen in pigment solutions. Photochem. Photobiol..

[60] Toftegaard R., Arnbjerg J., Daasbjerg K., Ogilby P.R., Dmitriev A., Sutherland D.S., Poulsen L. (2008). Metal-enhanced 1270 nm singlet oxygen phosphorescence. Angew. Chem. Int. Ed..

[61] Baker A., Kanofsky J.R. (1991). Direct observation of singlet oxygen phosphorescence at 1270 nm from L1210 leukemia cells exposed to polyporphyrin and light. Arch. Biochem. Biophys..

[62] Pang E., Zhao S., Wang B., Niu G., Song X., Lan M. (2022). Strategies to construct efficient singlet oxygen-generating photosensitizers. Coord. Chem. Rev..

[63] Chen T., Zheng Y., Gao Y., Chen H. (2022). Photostability investigation of a near-infrared-II heptamethine cyanine dye. Bioorg. Chem..

[64] Chinna Ayya Swamy P., Sivaraman G., Priyanka R.N., Raja S.O., Ponnuvel K., Shanmugpriya J., Gulyani A. (2020). Near infrared (NIR) absorbing dyes as promising photosensitizer for photo dynamic therapy. Coord. Chem. Rev..

[65] Medeiros N.G., Braga C.A., Câmara V.S., Duarte R.C., Rodembusch F.S. (2022). Near-infrared fluorophores based on heptamethine cyanine dyes: From their synthesis and photophysical properties to recent optical sensing and bioimaging applications. Asian J. Org. Chem..

[66] Moniruzzaman M., Dutta S.D., Lim K.-T., Kim J. (2022). Perylene-derived hydrophilic carbon dots with polychromatic emissions as superior bioimaging and NIR-responsive photothermal bactericidal agent. ACS Omega.

[67] Manivasagan P., Kim J., Jang E.-S. (2022). Recent progress in multifunctional conjugated polymer nanomaterial-based synergistic combination phototherapy for microbial infection theranostics. Coord. Chem. Rev..

[68] Lv Z., Jin L., Gao W., Cao Y., Zhang H., Xue D., Yin N., Zhang T., Wang Y., Zhang H. (2022). Novel YOF-based theranostic agents with a cascade effect for NIR-II fluorescence imaging and synergistic starvation/photodynamic therapy of orthotopic gliomas. ACS Appl. Mater. Interfaces.

[69] Zong J., Peng H., Qing X., Fan Z., Xu W., Du X., Shi R., Zhang Y. (2021). pH-responsive pluronic F127–lenvatinib-encapsulated halogenated boron-dipyrromethene nanoparticles for combined photodynamic therapy and chemotherapy of liver cancer. ACS Omega.

[70] Naskar N., Liu W., Qi H., Stumper A., Fischer S., Diemant T., Behm R.J., Kaiser U., Rau S., Weil T. (2022). A carbon nanodot based near-infrared photosensitizer with a protein-ruthenium shell for low-power photodynamic applications. ACS Appl. Mater. Interfaces.

[71] Liu Y., Li Y., Koo S., Sun Y., Liu Y., Liu X., Pan Y., Zhang Z., Du M., Lu S. (2022). Versatile types of inorganic/organic NIR-IIa/IIb fluorophores: From strategic design toward molecular imaging and theranostics. Chem. Rev..

[72] Chen X., Han H., Tang Z., Jin Q., Ji J. (2021). Aggregation-induced emission-based platforms for the treatment of bacteria, fungi, and viruses. Adv. Healthc. Mater..

[73] Mitsunaga M., Ito K., Nishimura T., Miyata H., Miyakawa K., Morita T., Ryo A., Kobayashi H., Mizunoe Y., Iwase T. (2022). Antimicrobial strategy for targeted elimination of different microbes, including bacterial, fungal and viral pathogens. Commun. Biol..

[74] Jia S., Sletten E.M. (2022). Spatiotemporal control of biology: Synthetic photochemistry toolbox with far-red and near-infrared light. ACS Chem. Biol..

[75] Li B., Wang W., Song W., Zhao Z., Tan Q., Zhao Z., Tang L., Zhu T., Yin J., Bai J. (2021). Antiviral and anti-inflammatory treatment with multifunctional alveolar macrophage-like nanoparticles in a surrogate mouse model of COVID-19. Adv. Sci..

[76] Seghatchian J., Struff W.G., Reichenberg S. (2011). Main properties of the THERAFLEX MB-plasma system for pathogen reduction. Transfus. Med. Hemotherapy.

[77] Mundt J.M., Rouse L., Van den Bossche J., Goodrich R.P. (2014). Chemical and biological mechanisms of pathogen reduction technologies. Photochem. Photobiol..

[78] Wainwright M., Mohr H., Walker W.H. (2007). Phenothiazinium derivatives for pathogen inactivation in blood products. J. Photochem. Photobiol. B.

[79] Harris F., Chatfield L., Phoenix D. (2005). Phenothiazinium based photosensitisers–photodynamic agents with a multiplicity of cellular targets and clinical applications. Curr. Drug Targets.

[80] Floyd R.A., Schneider J.E., Dittmer D.P. (2004). Methylene blue photoinactivation of RNA viruses. Antivir. Res..

[81] Tardivo J.P., Del Giglio A., de Oliveira C.S., Gabrielli D.S., Junqueira H.C., Tada D.B., Severino D., de Fátima Turchiello R., Baptista M.S. (2005). Methylene blue in photodynamic therapy: From basic mechanisms to clinical applications. Photodiagn. Photodyn. Ther..

[82] Yu S., Sun G., Sui Y., Li H., Mai Y., Wang G., Zhang N., Bi Y., Gao G.F., Xu P. (2021). Potent inhibition of severe acute respiratory syndrome coronavirus 2 by photosensitizers compounds. Dyes Pigm..

[83] Yano T., Minamide T., Takashima K., Nakajo K., Kadota T., Yoda Y. (2021). Clinical practice of photodynamic therapy using talaporfin sodium for esophageal cancer. J. Clin. Med..

[84] Sadraeian M., da Cruz E.F., Boyle R.W., Bahou C., Chudasama V., Janini L.M.R., Diaz R.S., Guimarães F.E.G. (2021). Photoinduced photosensitizer–antibody conjugates kill HIV env-expressing cells, also inactivating HIV. ACS Omega.

[85] Sadraeian M., Bahou C., da Cruz E.F., Janini L.M.R., Diaz R.S., Boyle R.W., Chudasama V., Guimarães F.E.G. (2020). Photoimmunotherapy using cationic and anionic photosensitizer-antibody conjugates against HIV env-expressing cells. Int. J. Mol. Sci..

[86] Zhang B., Zheng L., Huang Y., Mo Q., Wang X., Qian K. (2011). Detection of nucleic acid lesions during photochemical inactivation of RNA viruses by treatment with methylene blue and light using real-time PCR. Photochem. Photobiol..

[87] Steinmann E., Gravemann U., Friesland M., Doerrbecker J., Müller T.H., Pietschmann T., Seltsam A. (2013). Two pathogen reduction technologies–methylene blue plus light and shortwave ultraviolet light–effectively inactivate hepatitis C virus in blood products. Transfusion.

[88] Gendrot M., Andreani J., Duflot I., Boxberger M., Le Bideau M., Mosnier J., Jardot P., Fonta I., Rolland C., Bogreau H. (2020). Methylene blue inhibits replication of SARS-CoV-2 in vitro. Int. J. Antimicrob. Agents.

[89] Svyatchenko V.A., Nikonov S.D., Mayorov A.P., Gelfond M.L., Loktev V.B. (2021). Antiviral photodynamic therapy: Inactivation and inhibition of SARS-CoV-2 in vitro using methylene blue and radachlorin. Photodiagn. Photodyn. Ther..

[90] Ke M.-R., Eastel J.M., Ngai K.L.K., Cheung Y.-Y., Chan P.K.S., Hui M., Ng D.K.P., Lo P.-C. (2014). Photodynamic inactivation of bacteria and viruses using two monosubstituted zinc(II) phthalocyanines. Eur. J. Med. Chem..

[91] Mantareva V.N., Angelov I., Wöhrle D., Borisova E., Kussovski V. (2013). Metallophthalocyanines for antimicrobial photodynamic therapy: An overview of our experience. J. Porphyr. Phthalocyanines.

[92] Remichkova M., Mukova L., Nikolaeva-Glomb L., Nikolova N., Doumanova L., Mantareva V., Angelov I., Kussovski V., Galabov A.S. (2017). Virus inactivation under the photodynamic effect of phthalocyanine zinc(II) complexes. Z. Naturforsch. C.

[93] Ke M.-R., Eastel J.M., Ngai K.L.K., Cheung Y.-Y., Chan P.K.S., Hui M., Ng D.K.P., Lo P.-C. (2014). Oligolysine-conjugated zinc(II) phthalocyanines as efficient photosensitizers for antimicrobial photodynamic therapy. Chem. As. J..

[94] Zhou X., Zheng K., Li R., Chen Z., Yuan C., Hu P., Chen J., Xue J., Huang M. (2015). A drug carrier targeting murine uPAR for photodynamic therapy and tumor imaging. Acta Biomater..

[95] Korneev D., Kurskaya O., Sharshov K., Eastwood J., Strakhovskaya M. (2019). Ultrastructural aspects of photodynamic inactivation of highly pathogenic avian H5N8 influenza virus. Viruses.

[96] Sharshov K., Solomatina M., Kurskaya O., Kovalenko I., Kholina E., Fedorov V., Meerovich G., Rubin A., Strakhovskaya M. (2021). The photosensitizer octakis(cholinyl)zinc phthalocyanine with ability to bind to a model spike protein leads to a loss of SARS-CoV-2 infectivity in vitro when exposed to far-red LED. Viruses.

[97] Kamkaew A., Lim S.H., Lee H.B., Kiew L.V., Chung L.Y., Burgess K. (2013). BODIPY dyes in photodynamic therapy. Chem. Soc. Rev..

[98] Ziganshyna S., Szczepankiewicz G., Kuehnert M., Schulze A., Liebert U.G., Pietsch C., Eulenburg V., Werdehausen R. (2022). Photodynamic inactivation of SARS-CoV-2 infectivity and antiviral treatment effects in vitro. Viruses.

[99] Yuan A., Wu J., Tang X., Zhao L., Xu F., Hu Y. (2013). Application of near-infrared dyes for tumor imaging, photothermal, and photodynamic therapies. J. Pharm. Sci..

[100] Brilkina A.A., Dubasova L.V., Sergeeva E.A., Pospelov A.J., Shilyagina N.Y., Shakhova N.M., Balalaeva I.V. (2019). Photobiological properties of phthalocyanine photosensitizers Photosens, Holosens and Phthalosens: A comparative *in vitro* analysis. J. Photochem. Photobiol. B.

[101] Wang J., Zhao P., Li X., Fu H., Yang X., Wang G., Yang Y., Wei H., Zhou Z., Liao W. (2020). Evaluating the photodynamic biocidal activity and investigating the mechanism of thiazolium cyanine dyes. ACS Appl. Bio Mater..

[102] Frei A. (2020). Metal complexes, an untapped source of antibiotic potential?. Antibiotics.

[103] Frei A., Zuegg J., Elliott A.G., Baker M., Braese S., Brown C., Chen F., Dowson C.G., Dujardin G., Jung N. (2020). Metal complexes as a promising source for new antibiotics. Chem. Sci..

[104] Claudel M., Schwarte J.V., Fromm K.M. (2020). New antimicrobial strategies based on metal complexes. Chemistry.

[105] Evans A., Kavanagh K.A. (2021). Evaluation of metal-based antimicrobial compounds for the treatment of bacterial pathogens. J. Med. Microbiol..

[106] Nosaka Y., Daimon T., Nosaka A.Y., Murakami Y. (2004). Singlet oxygen formation in photocatalytic TiO_2_ aqueous suspension. Phys. Chem. Chem. Phys..

[107] Ma B.C., Ghasimi S., Landfester K., Zhang K.A.I. (2016). Enhanced visible light promoted antibacterial efficiency of conjugated microporous polymer nanoparticles *via* molecular doping. J. Mater. Chem. B.

[108] Posner G.H., Lever J.R., Miura K., Lisek C., Seliger H.H., Thompson A. (1984). A chemiluminescent probe specific for singlet oxygen. Biochem. Biophys. Res. Commun..

[109] Wu H., Song Q., Ran G., Lu X., Xu B. (2011). Recent developments in the detection of singlet oxygen with molecular spectroscopic methods. Tr. Anal. Chem..

[110] Pedersen S.K., Holmehave J., Blaikie F.H., Gollmer A., Breitenbach T., Jensen H.H., Ogilby P.R. (2014). Aarhus sensor green: A fluorescent probe for singlet oxygen. J. Org. Chem..

[111] Gollnick K., Griesbeck A. (1985). Singlet oxygen photooxygenation of furans. Tetrahedron.

[112] Ronzani F., Trivella A., Arzoumanian E., Blanc S., Sarakha M., Richard C., Oliveros E., Lacombe S. (2013). Comparison of the photophysical properties of three phenothiazine derivatives: Transient detection and singlet oxygen production. Photochem. Photobiol. Sci..

[113] Li L., Luo Z., Chen Z., Chen J., Zhou S., Xu P., Hu P., Wang J., Chen N., Huang J. (2012). Enhanced photodynamic efficacy of zinc phthalocyanine by conjugating to heptalysine. Bioconjugate Chem..

[114] Pişkin M. (2023). Phthalocyanine photosensitizers with bathochromic shift, of suitable brightness, capable of producing singlet oxygen with effective efficiency. J. Photochem. Photobiol. Chem..

[115] Sobotta L., Wierzchowski M., Mierzwicki M., Gdaniec Z., Mielcarek J., Persoons L., Goslinski T., Balzarini J. (2016). Photochemical studies and nanomolar photodynamic activities of phthalocyanines functionalized with 1,4,7-trioxanonyl moieties at their non-peripheral positions. J. Inorg. Biochem..

[116] Ferreira J.T., Pina J., Ribeiro C.A.F., Fernandes R., Tomé J.P.C., Rodríguez-Morgade M.S., Torres T. (2020). Highly efficient singlet oxygen generators based on ruthenium phthalocyanines: Synthesis, characterization and in vitro evaluation for photodynamic therapy. Chem. Eur. J..

[117] Santos P.F., Reis L.V., Almeida P., Oliveira A.S., Vieira Ferreira L.F. (2003). Singlet oxygen generation ability of squarylium cyanine dyes. J. Photochem. Photobiol. A.

[118] Atchison J., Kamila S., Nesbitt H., Logan K.A., Nicholas D.M., Fowley C., Davis J., Callan B., McHale A.P., Callan J.F. (2017). Iodinated cyanine dyes: A new class of sensitisers for use in NIR activated photodynamic therapy (PDT). Chem. Commun..

[119] Ciubini B., Visentin S., Serpe L., Canaparo R., Fin A., Barbero N. (2019). Design and synthesis of symmetrical pentamethine cyanine dyes as NIR photosensitizers for PDT. Dyes Pigm..

[120] Huang H., Huang D., Li M., Yao Q., Tian R., Long S., Fan J., Peng X. (2020). NIR aza-pentamethine dyes as photosensitizers for photodynamic therapy. Dyes Pigm..

[121] Štacková L., Muchová E., Russo M., Slavíček P., Štacko P., Klán P. (2020). Deciphering the structure–property relations in substituted heptamethine cyanines. J. Org. Chem..

[122] Cao J., Chi J., Xia J., Zhang Y., Han S., Sun Y. (2019). Iodinated cyanine dyes for fast near-infrared-guided deep tissue synergistic phototherapy. ACS Appl. Mater. Interfaces.

[123] Zhao X., Yao Q., Long S., Chi W., Yang Y., Tan D., Liu X., Huang H., Sun W., Du J. (2021). An approach to developing cyanines with simultaneous intersystem crossing enhancement and excited-state lifetime elongation for photodynamic antitumor metastasis. J. Am. Chem. Soc..

[124] Ma H., Lu Y., Huang Z., Long S., Cao J., Zhang Z., Zhou X., Shi C., Sun W., Du J. (2022). ER-targeting cyanine dye as an NIR photoinducer to efficiently trigger photoimmunogenic cancer cell death. J. Am. Chem. Soc..

[125] Santra M., Owens M., Birch G., Bradley M. (2021). Near-infrared-emitting hemicyanines and their photodynamic killing of cancer cells. ACS Appl. Bio Mater..

[126] Li Y., Ma T., Jiang H., Li W., Tian D., Zhu J., Li Z. (2022). Anionic cyanine J-type aggregate nanoparticles with enhanced photosensitization for mitochondria-targeting tumor phototherapy. Angew. Chem. Int. Ed..

[127] Ruan Z., Zhao Y., Yuan P., Liu L., Wang Y., Yan L. (2018). PEG conjugated BODIPY-Br_2_ as macro-photosensitizer for efficient imaging-guided photodynamic therapy. J. Mater. Chem. B.

[128] Batat P., Cantuel M., Jonusauskas G., Scarpantonio L., Palma A., O’Shea D.F., McClenaghan N.D. (2011). BF_2_-Azadipyrromethenes: Probing the excited-state dynamics of a NIR fluorophore and photodynamic therapy agent. J. Phys. Chem. A.

[129] Adarsh N., Avirah R.R., Ramaiah D. (2010). Tuning photosensitized singlet oxygen generation efficiency of novel aza-BODIPY dyes. Org. Lett..

[130] Miao X., Hu W., He T., Tao H., Wang Q., Chen R., Jin L., Zhao H., Lu X., Fan Q. (2019). Deciphering the intersystem crossing in near-infrared BODIPY photosensitizers for highly efficient photodynamic therapy. Chem. Sci..

[131] Karaman O., Almammadov T., Emre Gedik M., Gunaydin G., Kolemen S., Gunbas G. (2019). Mitochondria-targeting selenophene-modified BODIPY-based photosensitizers for the treatment of hypoxic cancer cells. ChemMedChem.

[132] Deckers J., Cardeynaels T., Penxten H., Ethirajan A., Ameloot M., Kruk M., Champagne B., Maes W. (2020). Near-infrared BODIPY-acridine dyads acting as heavy-atom-free dual-functioning photosensitizers. Chem. Eur. J..

[133] Bai J., Zhang L., Qian Y. (2021). A Near-infrared and lysosomal targeting thiophene-BODIPY photosensitizer: Synthesis and its imaging guided photodynamic therapy of cancer cells. Spectrochim. Acta. A..

[134] Ozketen A.C., Karaman O., Ozdemir A., Soysal I., Kizilenis C., Nteli Chatzioglou A., Cicek Y.A., Kolemen S., Gunbas G. (2022). Selenophene-modified boron dipyrromethene-based photosensitizers exhibit photodynamic inhibition on a broad range of bacteria. ACS Omega.

[135] Sun Y., Yu X., Yang J., Gai L., Tian J., Sui X., Lu H. (2021). NIR halogenated thieno[3,2-*b*]thiophene fused BODIPYs with photodynamic therapy properties in HeLa cells. Spectrochim. Acta. A..

[136] Yu Z., Wang H., Chen Z., Dong X., Zhao W., Shi Y., Zhu Q. (2022). Discovery of an amino acid-modified near-infrared aza-BODIPY photosensitizer as an immune initiator for potent photodynamic therapy in melanoma. J. Med. Chem..

[137] Gebremedhin K.H., Li M., Gao F., Gurram B., Fan J., Wang J., Li Y., Peng X. (2019). Benzo[a]phenoselenazine-based NIR photosensitizer for tumor-targeting photodynamic therapy via lysosomal-disruption pathway. Dyes Pigm..

[138] Tian M., Chen W., Wu Y., An J., Hong G., Chen M., Song F., Zheng W., Peng X. (2022). Liposome-based nanoencapsulation of a mitochondria-stapling photosensitizer for efficient photodynamic therapy. ACS Appl. Mater. Interfaces.

[139] Gourlot C., Gosset A., Glattard E., Aisenbrey C., Rangasamy S., Rabineau M., Ouk T.-S., Sol V., Lavalle P., Gourlaouen C. (2022). Antibacterial photodynamic therapy in the near-infrared region with a targeting antimicrobial peptide connected to a π-extended porphyrin. ACS Infect. Dis..

[140] Nagamaiah J., Dutta A., Pati N.N., Sahoo S., Soman R., Panda P.K. (2022). 3,6,13,16-Tetrapropylporphycene: Rational synthesis, complexation, and halogenation. J. Org. Chem..

[141] Wang J., Li J., Yu Z., Zhu X., Yu J., Wu Z., Wang S., Zhou H. (2022). Molecular tailoring based on Forster resonance energy transfer for initiating two-photon theranostics with amplified reactive oxygen species. Anal. Chem..

[142] Kim K.H., Kim S.J., Singha S., Yang Y.J., Park S.K., Ahn K.H. (2021). Ratiometric detection of hypochlorous acid in brain tissues of neuroinflammation and maternal immune activation models with a deep-red/near-infrared emitting probe. ACS Sens..

[143] Yang X., Bai J., Qian Y. (2020). The investigation of unique water-soluble heptamethine cyanine dye for use as NIR photosensitizer in photodynamic therapy of cancer cells. Spectrochim. Acta. A..

[144] Jiao L., Song F., Cui J., Peng X. (2018). A Near-infrared heptamethine aminocyanine dye with a long-lived excited triplet state for photodynamic therapy. Chem. Commun..

[145] Cai Z., Yu J., Hu J., Sun K., Liu M., Gu D., Chen J., Xu Y., He X., Wei W. (2023). Three near-infrared and lysosome-targeting probes for photodynamic therapy (PDT). Spectrochim. Acta. A..

[146] Yu Z., Zhou J., Ji X., Lin G., Xu S., Dong X., Zhao W. (2020). Discovery of a monoiodo aza-BODIPY near-infrared photosensitizer: In vitro and in vivo evaluation for photodynamic therapy. J. Med. Chem..

[147] Tian Y., Cheng Q., Dang H., Qian H., Teng C., Xie K., Yan L. (2021). Amino modified iodinated BODIPY photosensitizer for highly efficient NIR imaging-guided photodynamic therapy with ultralow dose. Dyes Pigm..

[148] Liu Y., Zhang Y., Liu G., Xing G. (2022). J- and H-aggregates of heavy-atom-free aza-BODIPY dyes with high ^1^O_2_ generation efficiency and photodynamic therapy potential. Dyes Pigm..

[149] Xing X., Yang K., Li B., Tan S., Yi J., Li X., Pang E., Wang B., Song X., Lan M. (2022). Boron dipyrromethene-based phototheranostics for near Infrared fluorescent and photoacoustic imaging-guided synchronous photodynamic and photothermal therapy of cancer. J. Phys. Chem. Lett..

[150] Jiang X.-D., Xi D., Le Guennic B., Guan J., Jacquemin D., Guan J., Xiao L.-J. (2015). Synthesis of NIR naphthyl-containing aza-BODIPYs and measure of the singlet oxygen generation. Tetrahedron.

[151] Jiang X., Zhang T., Sun C., Meng Y., Xiao L. (2019). Synthesis of aza-BODIPY dyes bearing the naphthyl groups at 1,7-positions and application for singlet oxygen generation. Chin. Chem. Lett..

[152] Liu Q., Tian J., Tian Y., Sun Q., Sun D., Wang F., Xu H., Ying G., Wang J., Yetisen A.K. (2021). Near-infrared-II nanoparticles for cancer imaging of immune checkpoint programmed death-ligand 1 and photodynamic/immune therapy. ACS Nano.

[153] Zou J., Li L., Zhu J., Li X., Yang Z., Huang W., Chen X. (2021). Singlet oxygen “afterglow” therapy with NIR-II fluorescent molecules. Adv. Mater..

[154] Zhao C., Wu B., Yang J., Baryshnikov G.V., Zhou Y., Ågren H., Zou Q., Zhu L. (2022). Large red-shifted NIR absorption in azulenyl- and iodinated-modified BODIPYs sensitive to aggregation and protonation stimuli. Dyes Pigm..

[155] Jun J.V., Chenoweth D.M., Petersson E.J. (2020). Rational design of small molecule fluorescent probes for biological applications. Org. Biomol. Chem..

[156] Ushio C., Ariyasu H., Ariyasu T., Arai S., Ohta T., Fukuda S. (2009). Suppressive effects of a cyanine dye against herpes simplex virus (HSV)-1 infection. Biomed. Res..

[157] Carpenter B., Situ X., Scholle F., Bartelmess J., Weare W., Ghiladi R. (2015). Antiviral, antifungal and antibacterial activities of a BODIPY-based photosensitizer. Molecules.

[158] Gattuso H., Marazzi M., Dehez F., Monari A. (2017). Deciphering the photosensitization mechanisms of hypericin towards biological membranes. Phys. Chem. Chem. Phys..

[159] Pourhajibagher M., Bahador A. (2020). Computational biology analysis of COVID-19 receptor-binding domains: A target Site for indocyanine green through antimicrobial photodynamic therapy. J. Lasers Med. Sci..

